# Extension of Tissue Plasminogen Activator Treatment Window by Granulocyte-Colony Stimulating Factor in a Thromboembolic Rat Model of Stroke

**DOI:** 10.3390/ijms19061635

**Published:** 2018-05-31

**Authors:** Ike C. dela Peña, Samuel Yang, Guofang Shen, Hsiao Fang Liang, Sara Solak, Cesar V. Borlongan

**Affiliations:** 1 Department of Pharmaceutical and Administrative Sciences, Loma Linda University, Loma Linda, CA 92350, USA; idelapena@llu.edu (I.C.d.P.); sjyang@llu.edu (S.Y.); gshen@llu.edu (G.S.); hliang@llu.edu (H.F.L.); ssolak@llu.edu (S.S.); 2 Center of Excellence for Aging and Brain Repair, Department of Neurosurgery and Brain Repair, University of South Florida Morsani College of Medicine, Tampa, FL 33612, USA; cborlong@health.usf.edu

**Keywords:** G-CSF, thromboembolic model, tPA, hemorrhagic transformation, vasculogenesis

## Abstract

When given beyond 4.5 h of stroke onset, tissue plasminogen activator (tPA) induces deleterious side effects in the ischemic brain, notably, hemorrhagic transformation (HT). We examined the efficacy of granulocyte-colony stimulating factor (G-CSF) in reducing delayed tPA-induced HT, cerebral infarction, and neurological deficits in a thromboembolic (TE) stroke model, and whether the effects of G-CSF were sustained for longer periods of recovery. After stroke induction, rats were given intravenous saline (control), tPA (10 mg/kg), or G-CSF (300 μg/kg) + tPA 6 h after stroke. We found that G-CSF reduced delayed tPA-associated HT by 47%, decreased infarct volumes by 33%, and improved motor and neurological deficits by 15% and 25%, respectively. It also prevented delayed tPA treatment-induced mortality by 46%. Immunohistochemistry showed 1.5- and 1.8-fold enrichment of the endothelial progenitor cell (EPC) markers CD34+ and VEGFR2 in the ischemic cortex and striatum, respectively, and 1.7- and 2.8-fold increases in the expression of the vasculogenesis marker von Willebrand factor (vWF) in the ischemic cortex and striatum, respectively, in G-CSF-treated rats compared with tPA-treated animals. Flow cytometry revealed increased mobilization of CD34+ cells in the peripheral blood of rats given G-CSF. These results corroborate the efficacy of G-CSF in enhancing the therapeutic time window of tPA for stroke treatment via EPC mobilization and enhancement of vasculogenesis.

## 1. Introduction

On average, one American has a stroke every 40 s, and one dies every 4 min [1]. Of the different types of stroke, acute ischemic stroke is the most common, and successful treatment of this medical condition remains very challenging. The “clot-busting” drug tissue plasminogen activator (tPA) is the only FDA drug approved for clinical use for acute ischemic stroke. However, treatment with the drug must be initiated within 4.5 h of stroke onset to avoid detrimental side effects, notably, hemorrhagic transformation (HT), which leads to high mortality in stroke patients [2]. Accordingly, an important clinical problem at hand is to develop methods that will extend the limited therapeutic time window of tPA or reduce the complications associated with delayed tPA treatment [3,4].

Granulocyte-colony stimulating factor (G-CSF), produced by the bone marrow stromal cells and fibroblasts, stimulates the proliferation, survival, and maturation of cells committed to the neutrophilic granulocyte lineage [5]. Exogenously administered G-CSF exerts neuroprotective effects in animal models of ischemia through a number of mechanisms, such as attenuation of glutamate-induced neurotoxicity, activation of the cerebral G-CSF receptor, and enhancement of angiogenesis and vasculogenesis attributable to G-CSF-induced activation and mobilization of bone marrow-derived endothelial progenitor cells (EPCs) [6,7,8,9,10,11,12]. Furthermore, we previously reported the efficacy of G-CSF in attenuating delayed tPA-induced HT and in improving post-stroke motor and neurological deficits in an intraluminal filament model of stroke [12]. These promising findings provide impetus for further exploration of the effects of G-CSF in attenuating delayed tPA-induced outcomes in other experimental stroke models, in line with the Stroke Therapy Academic Industry Roundtable (STAIR) guidelines for clinical development of drug candidates [13], and also for studies to determine the mechanisms of action and long-term effects of the drug, in order to enhance its future clinical application.

In keeping with our goal to pursue the development of G-CSF as a therapeutic agent to expand the limited therapeutic time window of tPA for ischemic stroke treatment, we conducted a follow-up study to examine whether the drug can reduce delayed tPA treatment-induced HT, worsening of neurological outcomes, and mortality in a thromboembolic (TE) model of stroke, a stringent model that closely mimics the clinical situation of vascular occlusion and reperfusion [14,15,16,17]. We also sought to determine whether G-CSF mobilized EPCs in the context of attenuating delayed tPA-induced HT possibly via vascular repair, and if the therapeutic effects of single G-CSF treatments were sustained at longer post-stroke time points.

## 2. Results

### 2.1. G-CSF Attenuated Delayed tPA-Induced Hemorrhage and Cerebral Infarction in a TE Stroke Model

**Figure 1:**
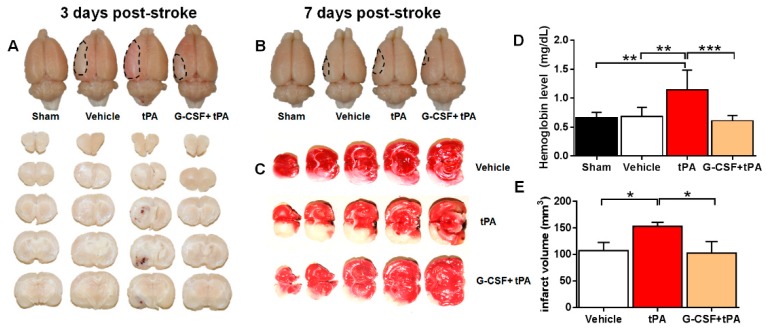
Effects of granulocyte-colony stimulating factor (G-CSF) on delayed tissue plasminogen activator (tPA)-induced hemorrhage and cerebral infarction in rats subjected to thromboembolic stroke. (**A**) Photographs are representative coronal brain sections obtained 3 days after stroke, showing that tPA treatment produced remarkable hemispheric hemorrhage, whereas the combined treatment with G-CSF did not result in bleeding; (**B**) Infarct volume was reduced, and hemorrhage was not observed in rats treated with G-CSF 7 days after stroke; (**C**) Representative coronal brain sections stained with 2,3,5-triphenyltetrazolium chloride (TTC) 3 days after stroke showing the infarct area (white) and intact areas (red); (**D**) Quantitative analysis of spectrophotometric assay findings showing the incidence of hemorrhage in rats subjected to delayed tPA treatment, which was reduced by 47% by G-CSF treatment; (**E**) Quantitative analysis of infarct volume in vehicle-, tPA-, and G-CSF + tPA-treated groups. G-CSF reduced infarct volume by 33% in stroked rats subjected to delayed tPA treatment; * *p* < 0.05, ** *p* < 0.01, *** *p* < 0.001, *n* = 7 rats per group. The data are expressed as mean ± S.E.M.

To examine whether G-CSF reduced delayed (i.e., 6 h after stroke) tPA-induced HT and cerebral infarction in a TE model, we evaluated hemorrhage volume and extent of cerebral infarction 3 days after stroke using spectrophotometric hemoglobin assay and 2,3,5-triphenyltetrazolium chloride (TTC) staining, respectively. Brain sections from the vehicle and G-CSF + tPA groups showed no detectable bleeding (Figure 1A). In contrast, brain sections obtained from tPA-treated rats displayed visible bleeding, indicating occurrence of HT following delayed tPA therapy. Representative rat brains obtained 7 days after stroke also portrayed HT in the tPA-treated rat, but not in vehicle-treated or G-CSF + tPA-treated subjects (Figure 1B). Quantitative analysis of cerebral hemorrhage revealed higher hemoglobin levels in tPA-treated rats compared with sham-treated rats (*t* (12) = 3.62, *p* < 0.01), indicating HT occurence (Figure 1D). Treatment with G-CSF in conjunction with tPA 6 h after stroke attenuated the HT due to delayed tPA treatment in stroked rats (*t* (12) = 4.03, *p* < 0.01) (Figure 1D). Meanwhile, rats in the tPA treatment group displayed a higher infarct volume compared with vehicle-treated animals (*t* (12) = 2.67, *p* < 0.05) (Figure 1C,E). Treatment with G-CSF reduced the increase in infarct volume (*t* (12) = 2.21, *p* < 0.05), indicating a cytoprotective effect of the drug (Figure 1E).

### 2.2. G-CSF Decreased Delayed tPA-Induced Neurological Deficits and Mortality

**Figure 2:**
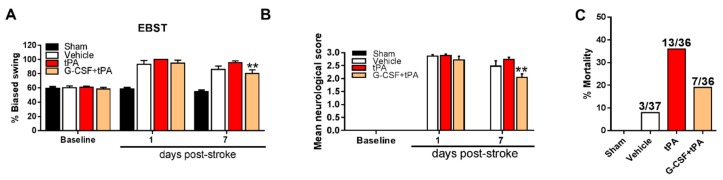
Effects of G-CSF on delayed tPA-induced motor and neurological deficits and mortality. (**A**) G-CSF treatment reduced delayed tPA-induced motor deficits by 15% as measured by the elevated body swing test (EBST); (**B**) G-CSF also reduced stroke and/or delayed tPA-induced neurological impairment by 25% when administered 7 days after stroke; (**C**) G-CSF decreased the incidence of mortality by 46% in stroke rats given tPA 6 h after stroke; ** *p* < 0.01 tPA vs. G-CSF + tPA, *n* = 10 rats per group. The data are expressed as mean ± S.E.M.

We conducted elevated body swing test (EBST) and neurological assessments to examine the effects of G-CSF on stroke and on delayed tPA-induced exacerbation of motor and neurological deficits. The tests were carried out prior to stroke induction and at 1 and 7 days after stroke. Two-way ANOVA of the EBST findings showed significant treatment (*F* (3, 36) = 35.46, *p* < 0.001) and day (*F* (2, 72) = 84.62, *p* < 0.001) effects and an interaction between treatment and days (*F* (6, 72) = 12.05, *p* < 0.001). Post-hoc testing demonstrated an improvement of stroke-induced motor deficits in G-CSF + tPA-treated rats compared with tPA-treated subjects 7 days after stroke (*p* < 0.01) (Figure 2A). Analysis of the neurological assessment findings revealed significant treatment (*F* (3, 36) = 304.2, *p* < 0.001) and day (*F* (2, 72) = 622.4, *p* < 0.001) effects and an interaction between treatment and days (*F* (6, 72) = 71.72, *p* < 0.001). Post-hoc tests detected significant improvement of stroke-induced neurological deficits in rats given G-CSF + tPA 7 days after stroke (*p* < 0.01), compared with rats subjected to tPA treatment only (Figure 2B). In light of the observation that delayed tPA treatment could result in HT, which could also lead to mortality in stroke patients, we evaluated whether G-CSF treatment reduced the mortality associated with delayed tPA therapy in stroke animals. The combined mortality rate (3 and 7 days after stroke, prior to sacrifice) due to delayed tPA treatment was decreased by 46% when G-CSF was administered to rats in conjunction with tPA (Figure 2C). 

### 2.3. Increased CD34+ and VEGFR-2 and vWF Expression in G-CSF+tPA-Treated Rats

**Figure 3:**
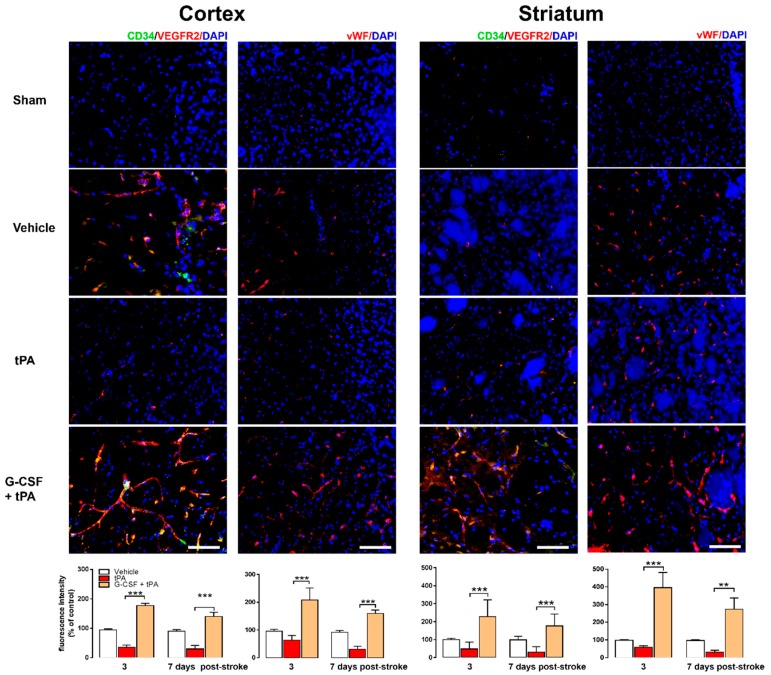
Immunohistochemical analyses of endothelial progenitor cell (EPC) markers CD34+ and VEGFR2 and of the vasculogenesis marker vWF in the ischemic cortex and striatum. Representative merged images obtained 3 days after stroke show co-localization of CD34+ and VEGFR2 or vWF with 4′,6-diamidino-2-phenylindole (DAPI; blue filter, nuclear staining). Analysis of fluorescence intensities showed that G-CSF treatment increased the expression of CD34+ and VEGFR2 in the ischemic cortex by 1.8- and 1.5-fold (vs. control) 3 and 7 days, respectively, after stroke. G-CSF also increased CD34+ and VEGFR2 expression in the ischemic striatum by 2.27- and 1.8-fold (vs. control) 3 and 7 days, respectively, after stroke. vWF expression was markedly increased by 2- and 1.7-fold (vs. control) in the ischemic cortex 3 and 7 days, respectively, after stroke. vWF expression was also enhanced by 4- and 2.8-fold (vs. control) in the ischemic striatum of G-CSF-treated animals 3 and 7 days, respectively, after stroke; ** *p* < 0.01, *** *p* < 0.001, *n* = 6 rats per group. The data are expressed as mean ± S.E.M. Horizontal bar: 100 μM.

The surface markers CD34+ and VEGFR-2 have been used extensively to characterize EPCs [18,19,20,21]. We examined the expression levels of these markers in the ischemic cortex and striatum in all rats 3 and 7 days after drug treatment and in the brains of the sham animals. Representative images obtained 3 days after stroke showed higher expression of CD34+ and VEGFR-2 in the ischemic cortex and striatum of G-CSF + tPA-treated rats compared with vehicle- and tPA-treated animals (Figure 3). Two-way ANOVA of CD34+ and VEGFR-2 total fluorescence intensity revealed significant effects of day (*F* (1, 15) = 6.45, *p* < 0.05) and treatment (*F* (2, 15) = 68.43, *p* < 0.001), but no interaction between day and treatment. Post-hoc tests revealed higher CD34+ and VEGFR-2 levels in the ipsilateral cortex of G-CSF + tPA-treated animals compared with tPA-treated rats 3 (*p* < 0.001) and 7 days (*p* < 0.001) after drug treatment. A significant treatment effect (*F* (2, 15) = 23.83, *p* < 0.001) was also found in the ipsilateral striatum of G-CSF+tPA-treated animals compared with tPA-treated rats 3 (*p* < 0.001) and 7 (*p* < 0.001) days after drug treatment. The von Willebrand factor (vWF) is a distinctive marker for vasculogenesis [18,22]. Representative images depicted higher vWF expression in the ischemic cortex and striatum of G-CSF + tPA-treated rats compared with vehicle- or tPA-treated animals on day 3 after drug treatment (Figure 3). Two-way ANOVA of vWF total fluorescence intensity showed a significant treatment effect (*F* (2, 15) = 17.42, *p* < 0.001), and post-hoc tests revealed higher vWF in the ipsilateral cortex of G-CSF+tPA-treated animals versus tPA-treated rats both 3 (*p* < 0.001) and 7 days (*p* < 0.001) after drug treatment. A notable treatment effect (*F* (2, 15) = 40.53, *p* < 0.001) was also found after comparing vWF fluorescence intensities in the ipsilateral striatum between G-CSF + tPA-treated rats and tPA-treated animals 3 (*p* < 0.001) and 7 days (*p* < 0.01) after drug treatment. The expression levels of the above-mentioned surface markers in the contralateral side of all rats (stroke and sham) did not vary significantly.

### 2.4. Increased CD34-Positive Cells in the Peripheral Blood of G-CSF+tPA-Treated Rats

**Figure 4:**
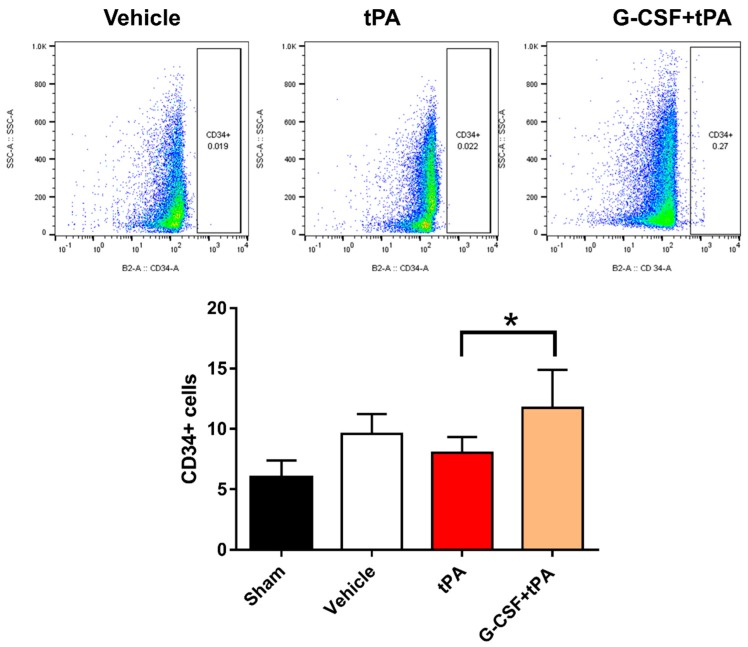
Mobilization of CD34+ cells in the peripheral blood by G-CSF. Top panel: Representative flow cytometry measurements of the number of CD34+ cells in the peripheral blood of vehicle-, tPA-, and G-CSF+tPA-treated stroked rats obtained 3 days after stroke. Isotype-matched antibodies were used as a control. Bottom: Quantitative analysis of the number of circulating CD34+ cells 3 days after stroke. G-CSF treatment significantly increased the number of CD34+ cells in the peripheral blood. The number of CD34+ cells in animals which did not undergo stroke surgery (sham) is also shown; * *p* < 0.05, *n* = 7–8 rats per group. The data are expressed as mean ± S.E.M.

That G-CSF can mobilize CD34+ EPC-containing bone marrow stem cells into the peripheral blood [23] indicates the potential role of G-CSF-mobilized EPCs in attenuating HT after delayed tPA treatment. We previously put forward the hypothesis that G-CSF diminishes delayed tPA-induced HT via induction of angiogenesis and vasculogenesis following an amplified activation of endothelial cells or G-CSF-mobilized EPCs [12]. One-way ANOVA of the flow cytometry data showed statistically significant differences between group means (*F* (3, 25) = 4.45, *p* < 0.05), and post-hoc tests revealed higher levels of CD34+ cells in the blood of rats injected with G-CSF + tPA compared to those given tPA only (*p* < 0.05) (Figure 4). This result indicates the mobilization of CD34+ EPC-containing bone marrow stem cells into the peripheral blood by G-CSF, in line with the observation that the drug exerts its therapeutic effects via activation or mobilization of CD34+ cells. 

## 3. Discussion

In accordance with the findings of our previous study [12], G-CSF attenuated the HT due to delayed (i.e., 6 h after stroke) tPA treatment in a thromboembolic rat model of stroke. Furthermore, G-CSF improved motor and neurological deficits 7 days after stroke, suggesting sustained neurologic and functional outcome improvements days after the initial G-CSF treatment. G-CSF also exerted neuroprotective effects, as evidenced by the reduction in infarct volume in stroke animals subjected to delayed tPA therapy. Importantly, G-CSF reduced the mortality due to delayed tPA administration, a remarkable effect of the drug in view of the clinical phenomenon of increased mortality risk with delayed tPA administration [2]. Immunohistochemistry revealed EPC activation and localization and enhanced vascularization in G-CSF-treated animals, as demonstrated by increased levels of EPC and vasculogenesis markers, CD34+ and VEGFR-2 and vWF, respectively, in the ipsilateral cortex and striatum of stroke animals. The flow cytometry finding of increased levels of CD34+ cells in the peripheral blood of rats injected with G-CSF lends further support to the recruitment and mobilization of EPCs by G-CSF. Taken together, the above observations substantiate the potentiality of G-CSF to reduce delayed tPA-induced HT and motor and neurological outcomes after stroke and to expand the limited therapeutic window of tPA. Notably, G-CSF combined with tPA was reported to enhance hemorrhage in experimental stroke [24]. However, it is difficult to compare the results of the present study with those of this previous study [24], given the differences in the experimental procedures (e.g., time of tPA administration, species of animals, mode of drug administration, G-CSF dose, etc.) and study objectives (enhancing thrombolysis versus reducing hemorrhage), as previously proposed [12,24]. 

Considering that up to 80% of human strokes are caused by thrombosis or embolism [14,15], we used the TE stroke model to examine the efficacy of G-CSF in attenuating delayed tPA-induced HT. Our findings add to the preclinical evidence supporting the potential use of G-CSF to reduce HT associated with delayed tPA therapy [12] and also address a STAIR guideline regarding efficacy assessments of drug candidates in multiple ischemia models prior to clinical development [13]. In the clinical scenario, delayed tPA treatment also increases the risk of fatal symptomatic hemorrhage [2,25]. We found that G-CSF reduced mortality, which could be an attractive feature of the drug if used as an adjunctive intervention to expand the limited treatment window of tPA for ischemic stroke. Moreover, we observed improvement of motor and neurological functions in G-CSF-treated animals 7 days after stroke, indicating lasting motor and behavioral effects of G-CSF treatment. To further enhance the potential utility of G-CSF to reduce delayed tPA treatment-induced outcomes and mortality, other preclinical assessments should be performed [13] in addition to the currently described procedure of testing the efficacy of G-CSF in different stroke models. For instance, since stroke afflicts mostly the elderly [26], it is important to test the efficacy of G-CSF in improving delayed tPA treatment-induced outcomes in old animals. In these animals, however, G-CSF must be given again a few days after the initial treatment, in view of the decreased regenerative capacity of the aged brain and the age-dependent reduction in neurogenesis and increased inflammatory response to stroke [26,27].

Some lines of evidence suggest that delayed tPA-induced HT is associated with detrimental effects of tPA on the neurovascular unit, characterized by disruption of the blood brain barrier (BBB), which is mainly comprised of endothelial cells [28]. Therefore, preserving the BBB by, e.g., preventing endothelial cell injury is a rational approach to counteract the HT due to delayed tPA reperfusion therapy after stroke [12]. We erstwhile demonstrated the reconstitution of the BBB mediated by EPCs after stroke [18], suggesting a potential role of EPCs in attenuating the delayed tPA-induced HT. EPCs, circulating cells that adhere to the endothelium at sites of hypoxia or ischemia, have been suggested to preserve the BBB through a number of mechanisms, including paracrine actions to enhance endothelial cell proliferation or stabilization [8,20,21]. In addition to tissue injury, G-CSF is known to mobilize EPCs [23], which have the capacity to subsequently differentiate into endothelial cells [29,30,31]. The immunohistochemistry findings of increased CD34+ and VEGFR-2 expression in the ischemic cortex and striatum of G-CSF-treated animals support the notion that G-CSF facilitates the homing of EPCs to ischemic sites. Moreover, the flow cytometry findings of increased CD34+ cells in the peripheral blood of G-CSF-treated rats signify recruitment or mobilization of EPCs after G-CSF treatment and provide another therapeutic implication for G-CSF-mobilized EPCs, i.e., in the attenuation of the delayed tPA-induced HT. Therefore, it is likely that G-CSF reduced the delayed tPA-induced HT via mobilization of EPCs, which localized to the ischemic site and maintained the integrity of the BBB by transforming into endothelial cells, or via direct actions of the drug, i.e., the preservation of endogenous endothelial cells [12]. However, other mechanisms may also be involved in view of the multiple neuroprotective actions of the drug [6,7,8,9,10,11,12]. Moreover, considering the role of inflammation in the pathogenesis of stroke [32,33] as well as, potentially, in the HT following delayed tPA treatment [34], it would be worthwhile to examine whether G-CSF alters the expression of inflammatory markers or of their modulators to reduce delayed tPA-induced outcomes, especially, HT. 

Vascularization, a reparative mechanism associated with the development of blood vessels and the improvement of tissue microperfusion around the ischemic boundary zone, promotes neuroprotection and improves functional outcomes after stroke [8,18]. Increased vascularization, especially in brain areas associated with motor and neurological functions (i.e., cortex and striatum), may underlie significant neuroprotection and behavioral improvements after stroke [8,18]. The increased vWF expression in the ischemic cortex and striatum of G-CSF-treated rats 3 and 7 days after stroke suggest enhanced vascularization in these brain regions, which could explain the motor and functional recovery in these animals. Although vascularization may require several days to complete, G-CSF might accelerate the process through mobilization of EPCs or preservation of endogenous endothelial cells, allowing the preservation of a patent vasculature not only to prevent delayed tPA-induced HT but also to produce neurologic and functional outcome improvements after stroke [3,4]. Of note, EPCs and other stem and progenitor cells are also known to participate in vasculogenesis [22]. Nevertheless, since the vasculature is also a source of cells that contribute to scar formation in the lesioned area [35], which could, therefore, impede structural and functional recuperation after stroke, other processes alongside the above-mentioned neovascularization could also contribute to the G-CSF-induced improvements in neurological and behavioral functions in stroke rats subjected to delayed tPA treatment [6,7,8,9,10,11,12].

In summary, using a TE model of stroke, we demonstrated the attenuation of delayed tPA-induced HT and neurological and functional outcome improvements in stroke animals that were administered G-CSF. Our results also indicate a key participation of G-CSF-mobilized EPCs in mediating the above-mentioned effects of G-CSF, consistent with our previous findings in the intraluminal filament stroke model [12]. Moreover, adding to the existing knowledge, we found other important effects of the drug, including reduction of mortality, sustained neurologic and functional outcome improvements days after the first G-CSF treatment, and recruitment of circulating EPCs, which are meaningful functions of the drug when viewed in the context of attenuating delayed tPA-induced HT. Therefore, this follow-up study provides additional evidence supporting the therapeutic potential of G-CSF to attenuate HT and other detrimental effects of delayed tPA therapy and, thus, to extend the limited therapeutic time window of tPA treatment for ischemic stroke. 

## 4. Materials and Methods

### 4.1. Animals

All experiments were performed in accordance with the Public Health Service Policy on Humane Care and Use of Laboratory Animals, the Guide for the Care and Use of Laboratory Animals, and other approved guidelines of the Loma Linda University Institutional Animal Care and Use Committee (IACUC#8150051; 29 January 2016). The procedures, data analysis, and reporting were carried out in accordance with the Animal Research: Reporting In Vivo Experiments guidelines (available online: https://www.nc3rs.org.uk/arrive-guidelines). Male adult Sprague–Dawley rats (approximately 9–10 weeks old) (Harlan Sprague Dawley, Indianapolis, IN, USA), weighing 200–250 g at the beginning of experiments were used for this study. They were housed in pairs in an AAALAC-approved Research Animal Facility, in a temperature- and humidity-controlled room maintained on 12 h light–dark cycles, with free access to food and water. In order to maintain gender consistency in our long-standing interest over the past two decades in testing stroke therapeutics, the study focused on male rats. Subsequent studies are planned in female rats following the demonstration of treatment safety and efficacy in males.

### 4.2. Study Design and Treatment Groups

After acclimatization, rats underwent sham (entire surgical procedure except injection of blood clots into the arteries) or stroke surgery (see below), and stroke rats were administered intravenously (via the tail vein) vehicle (0.9% saline), tPA (10 mg/kg) (a generous gift from Genentech, San Francisco, CA, USA), or G-CSF (300 µg/kg) (Amgen, Thousand Oaks, CA, USA) + tPA (10 mg/kg) 6 h post stroke [12]. The animals were randomly assigned to the experimental groups. The doses of tPA and G-CSF were determined in light of our previous findings [12]. One day and 7 days after drug treatment, the rats underwent behavioral testing. Three or 7 days after drug treatment, the rats were euthanized, and their brains were harvested for further experiments. 

### 4.3. Thromboembolic (TE) Stroke

Embolus preparation was performed according to the methods described by Zhang et al. [17]. In brief, femoral arterial blood was collected into a section of PE-50 tubing, and the blood-containing tubes were placed in a Petri dish at 37 °C for 2 h, followed by 22 h at 4 °C. A section of the clot was cut and transferred to a PE-10 tubing which was repeatedly rinsed with saline to flush out the erythrocytes. Four-cm fibrin-rich clots used for subsequent studies were collected into a modified PE-50 catheter connected to a 100 µL syringe and injected at the origin of the middle cerebral artery (MCA). During the surgery, the animals were anesthetized by a mixture of 1 to 2% isoflurane in NO/oxygen (69%/30%) via a face mask. The body temperature was maintained at 37 ± 0.3 °C during the surgical procedures. A midline skin incision was made in the neck of the animals with subsequent exploration of the right common carotid artery, the external carotid artery (ECA), and the internal carotid artery (ICA). Thereafter, a partial arteriotomy on the ECA was created, and the tip of a clot-filled modified PE-50 catheter was inserted into the arteriotomy and further advanced within the ICA rostrally, to enter the intracranial segment of the ICA until resistance was felt. Thereafter, the catheter was retracted, and the clot was slowly injected with 5–10 µL of saline at a rate of 10 µL/min. The cathether was retracted 5 min after clot delivery until its tip reached the ECA–ICA bifurcation. We have standardized the TE model in rats, with stroke animals showing ≥80% reduction in regional cerebral blood flow (CBF) during the occlusion period, as determined by laser Doppler (Perimed, Ardmore, PA, USA) [12]. We also found no significant differences in physiological parameters, including PaO_2_, PaCO_2_, and plasma pH measurements, in our stroke animals, indicating similar degree of stroke insults. Rats that reached the ≥80% CBF reduction during occlusion were used for the present studies.

### 4.4. Measurement of Brain Hemorrhage and Infarction

Hemorrhagic transformation was quantified using the spectrophotometric assay of brain hemoglobin content described in our previous study [12]. To measure infarct volume, coronal sections of the rats’ brains were stained with 2% 2,3,5-triphenyltetrazolium chloride (TTC) (Sigma, St. Louis, MO, USA), fixed with 4% paraformaldehyde, scanned, and analyzed to determine the ratio of infarct area to the whole brain, using ImageJ software (ImageJ2, Bethesda, MD, USA).

### 4.5. Motor and Neurological Tests

The animals were subjected to the elevated body swing test (EBST) and neurological examination before (baseline) stroke surgery and then at days 1 or 7 days after stroke. The procedures of the EBST are described in our previous studies [12,18]. To measure changes in neurological functions, we obtained neurological scores for each rat using three tests, namely, forelimb akinesia, beam walking ability, and paw grasp. The scores from these tests were pooled to obtain the mean neurological score for each treatment group. A ≥2.5 mean neurological score indicates stroke-induced neurological impairment. All behavioral tests were conducted by a single trained rater, blinded to all experimental conditions.

### 4.6. Histology and Immunohistochemistry

The rats were anesthetized and perfused transcardially with 4% paraformaldehyde in PBS. Their brains were removed and sliced into 40 μm sections in a cryostat and stored at −20 °C. Slide-mounted sections were incubated overnight at 4 °C with antibodies against the endothelial progenitor cell (EPC) markers CD34+ (15 µg/mL; R & D Systems, Minneapolis, MN, USA) and vascular endothelial growth receptor (VEGFR)-2 (1:100, Cell Signaling, Danvers, MA, USA) or the vasculogenesis marker von Willebrand factor (vWF; 1:100; Abcam, Cambridge, MA, USA) in PBS. After washing and incubation with secondary antibodies, the sections were washed and incubated for 30 min with Hoecst (ThermoFisher Scientific, Waltham, MA, USA) at 37 °C. After washing, the sections were mounted on coverslips using Fluoromount mounting medium. Control studies included the exclusion of primary antibodies, substituted with 5% normal goat serum in PBS. No immunoreactivity was observed in these controls.

### 4.7. Flow Cytometry

The rats were anesthetized, and blood was collected from their hearts. Thereafter, peripheral blood mononuclear cells were harvested from rat whole blood samples and stained with a PE-conjugated CD34 antibody (Abcam) in PBS for 30 min at 4 °C. The cells were then washed and stained with Fixable Viability eFluor 450 (eBiosciences, Waltham, MA, USA) to distinguish between living and dead cells. After washing, the cells were fixed with 1% PFA before analysis using the MACSQuant Analyzer (Miltenyi Biotech, San Diego, CA, USA). The appropriate isotype control was used. Data analysis was performed using FlowJo data analysis software (Ashland, OR, USA).

### 4.8. Statistical Analysis

We determined each sample size by power analysis using a significance level of α = 0.05 with 80% power to detect statistical differences. On the basis of our previous experience with similar experiments [12,18], *n* = 6 or more data points are generally needed for each group in order to obtain reliable results. All data are expressed as mean ± standard error of the mean (S.E.M.). The results were analyzed statistically using one- or two-way ANOVA and subsequent post-hoc Tukey’s tests, or unpaired *t*-tests when comparing the means of two groups. The data were analyzed by investigators blinded to the experimental treatments. Statistical significance was set at *p* < 0.05. All statistical analyses were conducted using GraphPad Prism 5 (San Diego, CA, USA).

## Abbreviations

G-CSF	granulocyte-colony stimulating factor
HT	hemorrhagic transformation
tPA	tissue plasminogen activator
EPC	endothelial progenitor cell
CD34	cluster of differentiation 4
VEGFR	vascular endothelial growth factor receptor
vWF	von Willebrand factor
